# Morpho-Physiological and Biochemical Criteria of *Acanthamoeba* spp. Isolated from the Egyptian Aquatic Environment

**Published:** 2013

**Authors:** A Al-Herrawy, M Bahgat, A Mohammed, A Ashour, W Hikal

**Affiliations:** 1 Parasitology Laboratory, Water Pollution Research Department, NRC, 12622 Dokki, Giza, Egypt; 2 Therapeutic Chemistry Department, NRC, 12622 Dokki, Giza, Egypt; 3 Zoology Department, Faculty of Science, Ain Shams University, Cairo, Egypt

**Keywords:** *Acanthamoeba*, Heat-tolerance, Proteases, Water, Swimming pools, Egypt

## Abstract

**Background:**

The free-living amoebae *Acanthamoeba* spp., have been recognized as etiologic agents of amoebic encephalitis, keratitis, otitis, lung lesions and other skin infections mainly in immuno-compromised individuals. In this study, morpho-physiological and biochemical characterization of *Acanthamoeba* strains isolated from the Egyptian aquatic environment were surveyed.

**Methods:**

Some *Acanthamoeba* species were cultivated on non-nutrient agar. Isolated strains of *Acanthamoeba* were identification based on the morphology of trophic and cyst forms in addition to temperature and osmo-tolerance assays. Biochemical characterization of the isolated amoeba strains was performed using quantitative assay as well as qualitative determination of proteolytic activity in zymograph analysis.

**Results:**

Potentially pathogenic *Acanthamoeba* species were isolated from all of the examined water sources. Colorimetric assays showed protease activity in the heat-tolerant isolates of *Acanthamoeba*. All pathogenic isolates of *Acanthamoeba* exhibited higher protease activity than did the non-pathogenic ones. The zymographic protease assays showed various banding patterns for different strains of *Acanthamoeba*.

**Conclusion:**

The incidence and prevalence of the pathogenic *Acanthamoeba* species in the aquatic environment using parasitological and biochemical diagnostic tools will provide baseline data against which the risk factors associated with waterborne transmission can be identified.

## Introduction

There is an increasing interest and awareness of the free-living amoeba, *Acanthamoeba*, over recent years as an opportunistic pathogen of medical importance. *Acanthamoeba* is one of the more abundant protozoa on earth, which can be isolated from soil, dust, air, treated and untreated tap water, swimming pools, air-conditioning units and numerous other domestic and outdoor environments (1). The protozoa's life cycle consists of an active feeding trophozoite phase and dormant cyst phase, which is activated by unfavorable conditions, such as exposure to extreme temperature, high or low pH, dryness or starvation. They are the causative agents of granulomatous amebic encephalitis and amebic keratitis and have been associated with cutaneous lesions and sinusitis. Human diseases caused by *Acanthamoeba* continue to rise worldwide, which is probably due to increasing populations of contact lens wearers and HIV+ patients (2). Moreover, *Acanthamoeba* serves as a carrier for different pathogens viruses, bacteria such as *Legionella*, *Pseudomonas* and *Helicobacter* (3). These bacterial pathogens can lead to severe human disease or manifest as complications of amoebic keratitis. Indeed, these amebas can transfer different microorganisms to humans and to date, *Acanthamoeba* is introduced as a vehicle for circulation of pathogens between human and environment (4).

The first human infection by *Acanthamoeba* was described causing granulomatous amoebic encephalitis (GAE) (5). In Egypt, *Acanthamoeba* spp. were isolated from the majority of environmental samples (70.27%).They were isolated from swimming pools, surface water and canals, River Nile, rain water, soil and air in different Egyptian governorates (6–9). The author reported the growth of the isolated *Acanthamoeba* spp. at 37 °C but not at 43 °C. Water samples from aquatic sites were examined for the presence of free living amoeba. In Lower Egypt, *A. culbertsoni*, *A. rhysodes* and *A. glebae* were isolated. Five *Acanthamoeba spp*. were isolated from Mahmoudia and Nubaria canals, which are the main water supply in Alexandria. These were *A. rhysodes*, *A. glebae*, *A. culbertsoni*, *A. astronyxis* and *A. palastiniensis* (10). *Acanthamoeba polyphaga* was isolated from the nasal swab of healthy children (11, 12).

The aim of this study was to determine the presence of *Acanthamoeba* spp. in different aquatic environment of Egypt. This finding lead to additional researches for investigating presence of pathogenic *Acanthamoeba* strains in water sources using morpho-physiological, and biochemical characterization methods which can be a risk factor for people especially contact lens wearers and immuno-compromised patients.

## Materials and Methods

### Samples and sampling sites

Water samples (3 liters each) were collected from different localities in Delta region, Egypt (Table 1) for the detection and isolation of freshwater amoebae. Samples were collected from the Nile River, tap water and swimming pool water in clean, dry autoclavable polypropylene containers and sent to the laboratory in icebox and processed at the same day of collection.

**Table 1:** Samples and sampling sites

Governorate	Type of water samples
Cairo	Nile, swimming pools and tap
Giza	Nile and tap
Qalubeya	Nile and tap
Behera	Nile and tap
Gharbeya	Nile and tap
Dakahleya	Nile and tap
Helwan	Nile, swimming pools and tap
Kafr-Elshikh	Tap
Sharkeya	Tap
Minofeya	Tap

### Isolation and morphologic identification of Acanthamoeba spp. from water samples

Collected water samples (1 liter each) were concentrated by using the membrane filtration technique. One liter of each water sample was filtered through a nitrocellulose membrane filters (0.45 µm pore size and 47 mm in diameter) (Whatman, WCN type, Cat No. 7141-104) (13). After filtration the membranes were separately inverted face to face on the surface of a non-nutrient (NN) agar plates previously seeded with 100 µl *Escherichia coli* suspension. All the inoculated plates were incubated at 40°C for one week with daily microscopic examination for the presence of any amoebic growth (14). Identification of the obtained *Acanthamoeba* spp. were achieved according to the morphological characteristics of both trophic and cyst stages (6, 15, 16).

### Osmo-tolerance assay for pathogenicity of isolated Acanthamoeba strains

The isolated free-living amoebae that proved morphologically to be *Acanthamoeba* were cultivated on non-nutrient agar plates containing one molar (1 M) manitol. Approximately 5 ml of late log phase cultures of *Escherichia coli* were poured on non-nutrient agar plates containing 1 M manitol and left for 5 min, after which the excess bacterial culture fluid was removed and plates were left to dry for 10 min. After that, 50 µl of each *Acanthamoeba* strain were separately inoculated at the center of the plate. Inoculated plates were incubated at 30°C for up to 72 hours with daily observation. Growth and persistence of pathogenic *Acanthamoeba* spp. was observed by measuring the increase in diameter of clearance zone in the bacterial lawn. The increase in zone diameter was an indication of the increase in pathogenicity of the inoculated *Acanthamoeba* spp. (17).

### Biochemical characterization of isolated Acanthamoeba *spp*.

Grown amoebae were harvested from cultured NN agar plates by scraping of the agar surface in eppendorf tubes containing 0.5 ml sterile Page's amoebae saline. The harvested amoebae were centrifuged at 1500 rpm for 10 min. The supernatant was discarded and the final pellet was re-suspended in 100 µl Page's amoebae saline and homogenized for 5 minutes in a tissue grinder. After that, the homogenate was transferred to a fresh eppendorf tube and centrifuged at 14000 rpm for 10 min. The supernatant was aspirated, divided into aliquots and stored at -80°C till being used. These steps were repeated for each sample (18).

### Quantitative assays for proteinase activity using chromomeric substrates

An aliquot was taken from samples prepared and stored at -80°C as mentioned above. The protease activities in different *Acanthamoeba* were quantitatively measured using the trypsin-like proteases specific substrate (Boc-Val-Leu-Gly-Arg-PNA L-1195, Bachem Biochemica, Heidelberg, Germany) at λmax 405 nm using Sun Rise reader (TECAN, Austria) according to (19–21). The intensity of the yellow colour was directly proportional to the enzyme activity.

### Qualitative determination of proteolytic activity in zymograph analysis (gelatin sodium dodecyl sulphate-polyacrylamide gel electrophoresis, SDS-PAGE gels)

The proteolytic activity of *Acanthamoeba* isolates were characterized by zymography on SDS-polyacrylamide gels copolymerized with gelatin (21).

### Statistical analysis

All obtained data were analyzed by the student's *t*-test using the Graph Pad InStat Soft ware.

## Results

### Prevalence of heat-tolerant *Acanthamoeba* spp. in different types of water

Heat-tolerant *Acanthamoeba* species were isolated from 56.0, 58.6 and 49.2% of the examined Nile water, tap water and swimming pools water samples, respectively.

Nile water samples collected from Helwan Governorate showed the highest incidence of heat-tolerant *Acanthamoeba* species (66.7%). The least incidence of *Acanthamoeba* species in Nile water samples occurred in Qalubeya (52.8%) and Gharbeya (38.9%) Governorates (Table 2). Tap water samples collected from Qalubeya Governorate showed the highest incidence of heat-tolerant *Acanthamoeba* species (88.9%). On the contrary, tap water of Gharbeya Governorate showed the lowest incidence of heat-tolerant *Acanthamoeba* species (41.7%) (Table 2). Water samples collected from swimming pools number 6 and 10 showed the highest incidence of heat-tolerant *Acanthamoeba* species (83.3%). Swimming pool number 2 recorded the least incidence of heat-tolerant *Acanthamoeba* species (25.0%). In addition, the heat-tolerant *Acanthamoeba* species were not recorded in water samples collected from swimming pool number 4 (Table 3).

**Table 2:** Prevalence of *Acanthamoeba* spp. in Nile and tap water samples

Sampling sites (Governorate)	Heat-tolerant *Acanthamoeba* grown at 40 °C					
	Nile water			Tap water		
	Examined samples (n)	+Ve samples	%	Examined samples (n)	+Ve samples	%
**Cairo**	36	21	58.3	36	18	50.0
**Giza**	36	20	55.6	36	17	47.2
**Qalubeya**	36	19	52.8	36	32	88.9
**Behera**	36	23	63.9	36	20	55.6
**Gharbeya**	36	14	38.9	36	15	41.7
**Dakahleya**	36	24	66.7	36	27	75.0
**Helwan**	36	20	55.6	36	22	61.1
**Kafr-Elshikh**	-	-	-	36	16	44.4
**Sharkeya**	-	-	-	36	28	77.8
**Minofeya**	-	-	-	36	16	44.4
**Total**	252	141	56.0	360	211	58.6

**Table 3:** Prevalence of *Acanthamoeba* spp. in swimming pool samples

Swimming pools	Examined samples (n)	Heat-tolerant free-living amoebae at 40°C *Acanthamoeba* spp.	
		+Ve	%
**1**	12	7	58.3
**2**	12	3	25.0
**3**	12	6	50.0
**4**	12	-	-
**5**	12	5	41.7
**6**	12	10	83.3
**7**	12	3	25.0
**8**	12	8	66.7
**9**	12	7	58.3
**10**	12	10	83.3
**Total**	120	59	49.2

### Osmo-tolerance differentiation between pathogenic and non-pathogenic Acanthamoeba

Results showed that pathogenic *Acanthamoeba* exhibit growth at increased osmolarity and this physiological determinant can be used to differentiate between pathogenic and non-pathogenic *Acanthamoeba*.

It was observed that 73 (51.8%) of 141 *Acanthamoeba* strains isolated from Nile water demonstrated pathogenic potential. Moreover, 23 of 211 (10.9%) *Acanthamoeba* strains isolated from tap water sources demonstrated pathogenic potential, but only 5 of 59 (8.5%) samples detected from swimming pools were considered as potentially pathogenic amoebae (Tables 4 and 5).

**Table 4:** Distribution of osmo-tolerant *Acanthamoeba* spp. in Nile and tap water samples

Sampling site (Governorate)	Tap water		Nile water	
	Osmo-tolerant +Ve samples/ *Acanthamoeba* +Ve samples			
	+Ve/ No.	%	+Ve/ No.	%
**Cairo**	4/18	22.2	12/21	57.1
**Giza**	2/17	11.8	12/20	60.0
**Qalubeya**	12/32	37.5	16/19	84.2
**Behera**	2/20	10.0	18/23	78.3
**Gharbeya**	0/15	0.0	3/14	21.4
**Dakahleya**	1/27	3.7	5/24	20.8
**Helwan**	2/22	9.1	7/20	35.0
**Kafr-Elshikh**	0/16	0.0	-	-
**Sharkeya**	4/28	14.3	-	-
**Minofeya**	5/16	31.3	-	-
**Total**	23/211	10.9	73/141	51.8

**Table 5:** Distribution of osmo-tolerant *Acanthamoeba* spp. in swimming pool samples

Swimming pool number	*Acanthamoeba* +Ve samples	Osmo-tolerant *Acanthamoeba* +Ve samples	
		No.	%
1	7	1	14.3
2	3	0	0.0
3	6	0	0.0
4	0	0	0.0
5	5	0	0.0
6	10	2	20.0
7	3	0	0.0
8	8	0	0.0
9	7	1	14.3
10	10	1	10.0
Total	59	5	8.5

### Morpho-physiological characteristics of isolated Acanthamoeba species

Identification of the different species of *Acanthamoeba* was performed according to the shape and size of cysts in addition to the number, shape, size and arrangement of the cyst pores. Six species of *Acanthamoeba* could be morphologically recognized, namely *Acanthamoeba castellanii*, *A. polyphaga*, *A. rhysodes, A*. *mauritaniensis, A. triangularis* and *A. royreba*.

### Quantitatively absolute enzyme activity in Acanthamoeba isolates

**Fig. 1:**
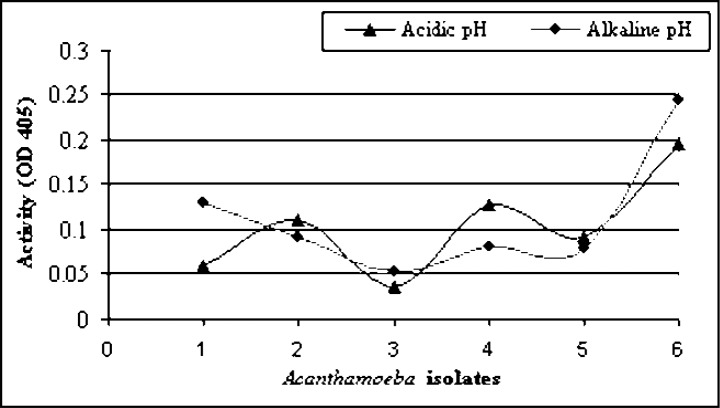
Tryptase activity in individual *Acanthamoeba* isolates at both acidic and alkaline pH

The examined *Acanthamoeba* isolates were classified according to absolute trypsin-like proteolytic activities into relatively pathogenic and non-pathogenic at both acidic and alkaline pH values (Fig. 1). *Acanthamoeba* isolates number 6 and 3 (isolated from swimming pools) showed the highest and lowest absolute trypsin-like activities at both acidic and alkaline pH values, respectively. These two *Acanthamoeba* isolates were morphologically identified as *A. polyphaga* and *A*. *triangularis*, respectively. Both of them exhibited thermo-tolerant activity. Concerning osmo-tolerance activity, the isolate number 6 exhibited growth at high osmolarity and is considered as potentially pathogenic while isolate number 3 exhibited no growth at high osmolarity and is considered as potentially non-pathogenic. Both *Acanthamoeba* isolates number 4, 5 and isolate number 2 (isolated from Nile water and tap water, respectively) showed moderately high proteolytic activities lying in between isolate number 6 and isolate number 3 at both acidic and alkaline pH values. They were morphologically identified as *A. rhysodes*, *A*. *royreba*,and *A. castellanii* respectively. Finally, isolate number 1 (isolated from tap water) showed moderately high proteolytic activity at alkaline pH value but low proteolytic activity at acidic pH value. This *Acanthamoeba* isolate, morphologically identified as *A. mauritaniensis*, exhibited both thermo- and osmo-tolerant properties and is considered as potentially pathogenic (Fig. 1).

### Qualitatively proteolytic activity in lysates of different Acanthamoeba isolates visualized by gelatin SDS-PAGE

**Fig. 2:**
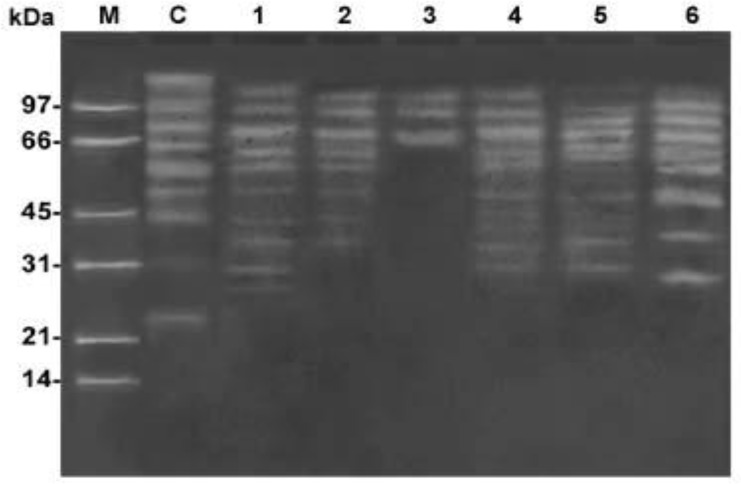
Serine-like protease activity in *Acanthamoeba* isolates was visualized in gelatin SDS-PAGE, where C: negative control bacteria; 1: *A. mauritaniensis*; 2: *A. castellanii*; 3: *A. triangularis*; 4: *A. rhysodes*; 5: *A. royreba*; 6: *A. polyphaga*; M: marker

The proteolytic profile of prepared lysates from bacterial control sample containing no *Acanthamoeba* was totally different from that of different *Acanthamoeba* isolates. Bacterial control sample had molecular weights 147, 110, 87, 70, 59, 53, 46, 31 and 23 kDa. The proteolytic profile of prepared lysates from *Acanthamoeba* isolates showed activity only at alkaline pH value 10, while at the acidic pH values no proteolytic activities appeared. Isolates number 1 and 2 (isolated from tap water and morphologically identified as *A*. *mauritaniensis* and *A. castellanii*, respectively) showed different proteolytic activities with common bands at molecular weights 63, 51, 43 and 36 kDa. The proteolytic activities of *A*. *mauritaniensis* were visualized at 118, 94, 75, 63, 57, 51, 43, 36, 30 and 27 kDa, while those of *A. castellanii* were visualized at 115, 93, 73, 63, 58, 51, 43 and 36 kDa (Fig. 2). *Acanthamoeba* isolates number 3 and 6 (isolated from swimming pools and morphologically identified as *A*. *triangularis* and *A. polyphaga*, respectively) showed completely different proteolytic activities. Only three proteolytic bands were seen at 115, 94 and 70 kDa in isolate number 3 (*A*. *triangularis*), while isolate number 6 (*A. polyphaga*) the gelatin digestion bands were evidenced at 104, 87, 71, 61, 57, 48, 34 and 29 kDa (Fig. 2).

The proteolytic activity from isolates number 4 and 5 (isolated from Nile water, morphologically identified as *A. rhysodes* and *A*. *royreba*, respectively) showed different proteolytic activities with a common band at molecular weight 30 kDa. The proteolytic activities of *A. rhysodes* were visualized at 113, 90, 75, 62, 49, 45, 39, 36 and 30 kDa, while in *A*. *royreba* the gelatin digestion bands were evidenced at 124, 97, 83, 73, 65, 61, 57, 50, 37 and 30 KDa (Fig. 2). The intensity of the bands varied among species. The pH profile revealed that these proteases were active in all strains at pH 10 only. Interestingly, these proteases showed over expression only in potentially pathogenic species of *Acanthamoeba*.

## Discussion

The present study deals with the natural distribution of pathogenic *Acanthamoeba* in the aquatic environment at different localities in the Nile Delta region, Egypt. Of special interest were the isolates capable of proliferating at temperatures of 37°C and above, as well as the biochemical characterization of these organisms. Previous studies on this subject in Egypt are non-existent. Therefore this study represents the first field investigation initiated in Egypt to estimate the presence of potentially pathogenic *Acanthamoeba* in the Nile, tap and swimming pools waters by using culture and biochemical techniques.

### Prevalence of heat tolerant free-living amoebae in different types of water

In the present study, free-living amoebae grown at 40°C were isolated from 78.9% of the total examined freshwater samples. In Oklahoma, USA, (22) recorded a relatively lower incidence of free-living amoebae (63.0%) isolated at 42°C from pond filled mainly by runoff water. A year later, the same authors recorded free-living amoebae in a much lower incidence (28.5%) when water samples from Lake Tenkiller were cultured at 42°C (23).

In the present work, free-living amoebae were isolated at 37°C from 90.5% of the examined tap water samples. In Egypt Hamadto et al. (7) and Hilali et al. (24) recorded lower incidence of free-living amoebae in tap water (4 and 23.6%, respectively). This difference might be due to the difference in the number of the samples examined: 50 and 72 tap water samples respectively, compared to 360 tap water samples in the present investigation. Certain workers in UK also detected free-living amoebae in 89% of the examined household tap-water samples (25). However, in Korea, Jeong and Yu (26) recorded freshwater amoebae in a percentage of domestic tap water samples (46.9%) lower than that recorded in our results. In the present study, free-living amoebae grown at 40°C were isolated from 60% of the swimming pool samples. In Poland, researchers (27) recorded a lower incidence of free-living amoebae (37.2%) isolated at 42°C from swimming pools.

### Prevalence of Acanthamoeba in different types of water

In the present study, it was found that the incidence of *Acanthamoeba* spp. reached 56.0% in the examined Nile water samples. In Egypt also *Acanthamoeba* spp. were detected in 66.67, 40 and 43.1% of the examined Nile water samples (6, 7, 24), respectively. Lorenzo-Morales et al. (8) in Spain detected *Acanthamoeba* in 43.3% of fresh water samples examined. Other workers in Thailand, USA and Saudi Arabia recorded a lower incidence of *Acanthamoeba* (35, 38.89 and 36.7%, respectively) in freshwater samples (22, 28, 29). In USA, Ettinger et al. (30) recorded *Acanthamoeba* spp. from James River in a percentage (7%) lower than that recorded in our results.

In the present work, it was found that the incidence of *Acanthamoeba* spp. reached 58.6% in the examined tap water samples. However, in Egypt Hamadto et al. (7) detected *Acanthamoeba* in tap water in a much lower incidence (4%). This difference might be due to the difference in the number of the examined samples as the mentioned authors examined 50 tap water samples; while in the present work a much higher number of samples (360) were examined. Other workers in Egypt detected *Acanthamoeba* in a lower percentage (11.1%) in the examined tap water (24) than that of our results. Other workers in other countries, in Spain for example, the incidence of *Acanthamoeba* in tap water recorded 59.5% (31). Other workers in other countries also recorded lower incidence of *Acanthamoeba* (26.9 and 5.8%) in tap water samples in UK and Korea, respectively (25, 26), for example.

In the present work, it was found that the incidence of *Acanthamoeba* spp. reached 49.2% in the examined swimming pool samples. However, in Egypt *Acanthamoeba* spp. were detected in a lower incidence (25%) of the examined swimming pool samples (7). Other workers in NewZeland, Mexico, Thailand, and Taiwan recorded lower incidences of *Acanthamoeba* (20, 32, 8.1, 13.0 and 5.9%, respectively) in swimming pools (32–36). These findings may indicate that the used disinfection procedures were not adequate for the elimination of amoebae from the swimming pools examined.

### Osmo-tolerance differentiation between pathogenic and non-pathogenic Acanthamoeba

*Acanthamoeba* spp. exhibiting growths at 1M manitol are considered potentially pathogenic. This physiological assay can be used to differentiate between pathogenic and non-pathogenic *Acanthamoeba* spp. (17, 37, 31). In the present work it was found that 92.9% of the isolated *Acanthamoeba* strains from Nile water demonstrated pathogenic potential using osmo-tolerance assay. Also in the present work, the percentage of *Acanthamoeba* spp. exhibiting osmo-tolerance reached 15.2 and 8.5% in tap water and swimming pools, respectively. Other workers in Egypt, recorded a lower incidence of osmo-tolerant *Acanthamoeba* spp. (69%) isolated from freshwater samples of the Nile Delta (8). Other workers in Turkey from fresh water samples reported a lower percentage of *Acanthamoeba* spp. exhibited osmo-tolerance (66.6%) (38).

### Biochemical Characterization of isolated free-living amoebae

Proteases are enzymes that catalyze the hydrolysis of peptide bonds in a broad spectrum of important biological reactions including the pathogenesis of parasitic disease (39). Proteases secreted by *Acanthamoeba* are regarded as an important factor in the pathogenesis (39).

In the present work, the isolated *Acanthamoeba* species were classified according both osmo-tolerant and enzymatic activity tests to 5 potentially pathogenic (morphologically identified as *Acanthamoeba rhysodes*, *A. polyphaga*, *A*. *mauritaniensis, A. royreba* and *A. castellanii*) and 1 non-pathogenic (morphologically identified as *A. triangularis*) species. Our result agreed with other workers in UK (18) in that *A. castellanii* exhibited significantly higher protease activity, while our result disagreed in that *A. polyphaga* and *A. royreba* (that were potentially pathogenic in our result) exhibited a lower protease activity. In the present work, *A. polyphaga* showed proteolytic activity ranging from 29 to 104 kDa by zymography. Other workers in Mexico (40) found that *A. polyphaga* gave proteolytic activity ranging from 34 to 144 kDa and they agreed with our results in 2 bands (34 and 71 kDa). Other workers reported that *A. polyphaga* zymography gave 43, 59, 70 and 100 to 130 kDa cysteine protease, (41) and our results also showed a dense band of 104 kDa. Mitro et al. (42) clearly showed only three protease activities of 36, 49 and 66 kDa, accompanied by additional faint bands that were discernible between 22 and 24 kDa and between 49 and 66 kDa.

In the present work, *A. castellanii* showed proteolytic activity ranging from 36 to 115 kDa by zymography. In Egypt, Nashed et al. (43) found complete agreement among the *A. culbertsonii* strains with respect to the banding patterns of phosphoglucomutase using disc electrophoresis of seven different enzymes to compare *A. culbertsonii* from Egypt with other *A. culbertsonii* from New York and India and *A. castellanii* from London. Other workers in Mexico (40) stated that *A. castellanii* gave a characteristic zymography bands ranging from 30 to 178 kDa but they reported a band giving 73 kDa which we recorded in the present work. On the other hand, *A. castellanii* was described as having different bands by zymography (44–47).

Other workers described that a characteristic band at 33 kDa was considered as an indicator of pathogenicity due to the presence of serine proteases in different *Acanthamoeba* spp. (44, 47–49). In the present study a 34 kDa band was only observed in *A. polyphaga* and 36 kDa was only in *A. castellanii*.

## Conclusion

The incidence and prevalence of the pathogenic *Acanthamoeba* species in the aquatic environment using parasitological and biochemical diagnostic tools will provide baseline data against which the risk factors associated with waterborne transmission can be identified.
